# Association of the US President’s Emergency Plan for AIDS Relief’s Funding With Prevention of Mother-to-Child Transmission of HIV in Kenya

**DOI:** 10.1001/jamanetworkopen.2019.11318

**Published:** 2019-09-13

**Authors:** Dale A. Barnhart, Isaac Tsikhutsu, Duncan Kirui, Fredrick Sawe, Jane Muli, William Sugut, Nareen Abboud, Deborah Birx, Tiffany Hamm, Peter Coakley, Patrick W. Hickey, Vanessa Wolfman, Elizabeth Lee, Donna Spiegelman

**Affiliations:** 1 Department of Epidemiology, Harvard T.H. Chan School of Public Health, Boston, Massachusetts; 2 Henry M. Jackson Foundation Medical Research International, Kericho, Kenya; 3 Office of the Global AIDS Coordinator and Health Diplomacy Department of State, Washington, DC; 4 Henry M. Jackson Foundation for the Advancement of Military Medicine Inc, Bethesda, Maryland; 5 US Military HIV Research Program, Walter Reed Army Institute of Research, Silver Spring, Maryland; 6 Department of Pediatrics, Uniformed Services University, Bethesda, Maryland; 7 Center for Methods in Implementation and Prevention Science, Department of Biostatistics, Yale School of Public Health, New Haven, Connecticut; 8 Department of Biostatistics, Harvard T.H. Chan School of Public Health, Boston, Massachusetts; 9 Department of Global Health, Harvard T.H. Chan School of Public Health, Boston, Massachusetts; 10 Department of Nutrition, Harvard T.H. Chan School of Public Health, Boston, Massachusetts

## Abstract

**Importance:**

From 2004 to 2014, the US President’s Emergency Plan for AIDS Relief (PEPFAR) invested more than $248 000 000 in the prevention of mother-to-child transmission (PMTCT) of HIV in Kenya. Concurrently, child mortality in Kenya decreased by half.

**Objective:**

To identify the extent to which the decrease in child mortality in Kenya is associated with PEPFAR funding for PMTCT of HIV.

**Design, Setting, and Participants:**

This population-based survey study conducted in Kenya estimated the association between annual per capita PEPFAR funding for PMTCT (annual PCF) and cumulative per capita PEPFAR funding for PMTCT (cumulative PCF), extracted using 2004-2014 country operational reports as well as individual-level health outcomes, extracted from the 2003, 2008-2009, and 2014 Kenya Demographic and Health Surveys and the 2007 and 2012 Kenya AIDS Indicator Surveys. The study included children of female respondents to the 2003, 2008-2009, and 2014 Kenya Demographic and Health Surveys who were born 1 to 60 months (for neonatal mortality) or 12 to 60 months (for infant mortality) before the survey, as well as female respondents who had recently given birth and reported on HIV testing during antenatal care (ANC) during the 2007-2014 surveys. Results were adjusted for year, province, and survey respondent characteristics. Statistical analysis was performed from July 8, 2016, to December 10, 2018.

**Main Outcomes and Measures:**

Neonatal mortality was defined as death within the first month of life and infant mortality was defined as death within the first year of life. HIV testing during ANC was defined as receiving counseling on PMTCT, undergoing an HIV test, and receiving test results during ANC.

**Results:**

The analysis included 33 181 neonates (16 870 boys), 26 876 infants (13 679 boys), and 20 775 mothers (mean [SD] age, 28.0 [6.7] years). PEPFAR funding was not associated with neonatal mortality. A $0.33 increase in annual PCF, corresponding to the difference between the 75th and 25th (interquartile range) percentiles of funding, was significantly associated with a 16% (95% CI, 4%-27%) reduction in infant mortality after a 1-year lag. A 14% to 16% reduction persisted after 2- and 3-year lags, and comparable reductions were observed for unlagged and 1-year lagged cumulative PCF. An increase of 1 interquartile range in cumulative PCF was associated with a 7% (95% CI, 3%-11%) increase in HIV testing during ANC, which intensified with subsequent lags. Between 2004 and 2014, sustained funding levels of $0.33 annual PCF could have averted 118 039 to 273 924 infant deaths.

**Conclusions and Relevance:**

Evidence from publicly available data suggests that PEPFAR’s PMTCT funding was associated with a reduction in infant mortality and an increase in HIV testing during ANC in Kenya. The full outcome of funding may not be realized until several years after allocation.

## Key Points

**Question:**

Is funding by the US President’s Emergency Plan for AIDS Relief for prevention of mother-to-child transmission of HIV associated with improvements in key prevention of mother-to-child transmission of HIV–related health outcomes in Kenya?

**Findings:**

Using publicly available data sources, this population-based survey study found evidence that a $0.33 increase in the annual levels of President’s Emergency Plan for AIDS Relief funding for prevention of mother-to-child transmission of HIV per capita was associated with a significant 14% to 16% reduction in infant mortality in Kenya between 2004 and 2014.

**Meaning:**

Identifying specific HIV prevention approaches, such as prevention of mother-to-child transmission of HIV, that are associated with improvements in health outcomes may help policy makers allocate funds to evidence-based approaches that are most likely to be effective.

## Teaser

This population-based survey study examines the extent to which the decrease in child mortality in Kenya is associated with the US President’s Emergency Plan for AIDS Relief (PEPFAR) funding for prevention of mother-to-child transmission of HIV.

## Introduction

Between 1988 and 2003, Kenya experienced a 32% increase in mortality among children younger than 5 years, which has been partially attributed to the HIV epidemic.^1,2^ In response, Kenya established prevention of mother-to-child transmission (PMTCT) of HIV programs in more than 10 000 facilities.^3^ This achievement was supported by the US President’s Emergency Fund for AIDS Relief (PEPFAR), which contributed more than $248 million to PMTCT programs in Kenya between 2004 and 2014.^4^

Although PEPFAR’s investments in PMTCT coincided with a halving of the mortality rate among children younger than 5 years in Kenya,^5^ it is unknown whether this improvement can be attributed to PEPFAR funding for PMTCT. Programs for PMTCT provide a series of interventions, including provision of HIV testing during antenatal care (ANC), prescription of antiretroviral medication to HIV-positive mothers and exposed infants, and counseling on safe breastfeeding practices, which are critical to the survival of children born to HIV-positive mothers.^6^ Without PMTCT, 25% to 48% of children born to HIV-positive mothers become HIV positive, and, in low-resource settings, 50% of HIV-positive children who do not receive treatment die before 2 years of age.^7,8^ However, child mortality decreased in most sub-Saharan African countries during the 2000s,^9^ and regional trends, rather than PEPFAR funding, could explain all or part of Kenya’s reductions in child mortality. In addition, PEPFAR-funded activities targeting adults could have displaced essential newborn and infant health services,^10,11^ resulting in worsened child health outcomes.

Although PEPFAR-focus countries have experienced greater reductions in adult mortality than nonfocus countries,^12,13^ most previous studies have not found corresponding reductions in child mortality.^11,14,15^ However, prior research has not specifically assessed the association of PEPFAR funding with PMTCT, one of PEPFAR’s activities most directly linked to children, and child health outcomes. In this study, we evaluated whether the amount of PEPFAR funding for PMTCT is associated with decreased probability of neonatal and infant mortality and increased probability of the receipt of HIV counseling, testing, and test results as part of ANC in Kenya.

## Methods

### Data Sources

Health outcome data came from the publicly available Kenya Demographic and Health Surveys and Kenya AIDS Indicator Surveys (KAIS), which use stratified 2-stage cluster random sampling to select nationally representative samples. Our analysis included data from the 5 most recent surveys: the 2003, 2008-2009, and 2014 Kenya Demographic and Health Surveys and the 2007 and 2012 KAIS.^3,5,16,17,18^ Neonatal mortality, defined as death within the first month of life, and infant mortality, defined as death within the first year of life, were assessed using female respondents’ birth histories from the 2003, 2008-2009, and 2014 surveys. For each live birth, mothers reported their child’s birth date, vital status, and death date, if applicable. Because maternal HIV is a common cause of maternal and infant death, birth histories can underestimate infant mortality in generalized HIV epidemics^19^; however, bias can be reduced by considering recent births.^20^ Therefore, neonatal mortality was assessed among children born 1 to 60 months prior to the interview date and infant mortality was assessed among children born 12 to 60 months prior to the interview date. Although the 2012 KAIS gathered data on abbreviated birth histories, we excluded it in our primary analysis because neonatal and infant mortality was not included in the final report of the survey (eAppendix and eTable 1 in the Supplement).^3^ Rates of HIV testing during ANC, which is one mechanism through which PMTCT programs can help prevent vertical transmission of HIV and subsequent child mortality, were measured among female respondents who had given birth within 5 years of the 2007, 2008-2009, and 2012 surveys and within 2 years of the 2014 survey and is defined as receiving counseling on PMTCT, undergoing an HIV test, and receiving test results during ANC. For women with multiple births, data on HIV testing during ANC were gathered for the most recent birth. Data on the independent variable, PEPFAR funding for PMTCT, were extracted from publicly available Country Operational Plans (COPs), which describe annual planned expenditures^21^ (eAppendix, eFigure 1, and eTable 2 in the Supplement).

This research involved the collection and analysis of preexisting, publicly available, deidentified data and received an exemption for review from the Harvard T.H. Chan School of Public Health Institutional Review Board and a “not research” designation from the US Army Medical Research and Materiel Command, Human Research Protection Office. The protocol was additionally reviewed and approved by the Kenya Medical Research Institute’s Scientific and Ethics Review Unit. This study followed the Strengthening the Reporting of Observational Studies in Epidemiology (STROBE) reporting guidelines. Additional details on the sampling frame, response rates, and other ethical and methodological considerations for the Kenya Demographic and Health Surveys and KAIS can be found elsewhere.^3,5,16,17,18^

### Statistical Analysis

Statistical analysis was performed from July 8, 2016, to December 10, 2018. We assessed the association between the amount of per capita PEPFAR funding for PMTCT and health outcomes using regression models.^22^ For annual per capita funding (annual PCF), each individual’s exposure equaled the amount of PEPFAR funding allocated to their province of residence in their year of birth (or, for HIV testing during ANC, in the year they gave birth) divided by the province’s population.^23^ For cumulative per capita funding (cumulative PCF), each individual’s exposure to PEPFAR funding was calculated using the cumulative total of PEPFAR funding for PMTCT allocated to their province from the beginning of PEPFAR until their year of birth. We investigated 0- to 3-year lags between funding allocation and the time that funding was hypothesized to have effects (eTable 2 in the Supplement). Individuals residing in province-years without funding data were excluded from the primary analyses but were included in sensitivity analyses.

We estimated risk ratios and 95% CIs associated with PEPFAR funding for PMTCT using weighted generalized estimating equations^24^ that created a representative sample across surveys and stepwise selection of restricted cubic splines that assessed for potential nonlinear associations between funding and health outcomes.^25^ All models treated individual birth as the unit of analysis and adjusted for province and controlled for calendar year using restricted cubic splines. To address confounding, we present both minimally adjusted models, which adjusted only for province and calendar year, and fully adjusted models, which included additional factors that were believed to be potentially important confounders that were known or potential risk factors for the outcomes. Fully adjusted models further controlled for household wealth quintile, water and sanitation access, urban vs rural status, mosquito net ownership, respondent educational level, ethnicity, religion, marital status, age, parity, and exposure to mass media, and, for neonatal and infant mortality, child’s sex, short interval preceding birth, and birth order rather than parity (eAppendix in the Supplement). To estimate the number of lives saved by PEPFAR funding for PMTCT, we used the 1-year lagged model to determine the number of infants who would have died between 2004 and 2014 under different levels of annual PCF (eAppendix in the Supplement).

We evaluated the sensitivity of our results to missing exposure data by conducting analyses assuming that province-years with missing funding data received $0.04 in annual PCF (reflecting the minimum observed funding level), $0.23 in annual PCF (reflecting the 25th percentile of observed funding levels), $0.32 in annual PCF (reflecting the median of observed funding levels), $0.56 in annual PCF (reflecting the 75th percentile of observed funding levels), and $0.93 in annual PCF (reflecting the maximum observed funding level) and, for infant mortality, using inverse probability weighting (eAppendix in the Supplement).^26^ We also assessed whether the association between PEPFAR funding and infant mortality differed by maternal HIV status among the subset of infants with known maternal HIV status based on HIV testing conducted in the 2003 and 2008 surveys (eAppendix in the Supplement).

## Results

### Survey Response

The 2003 survey included data on 8195 female respondents, the 2007 survey included data on 10 244 female respondents, the 2008-2009 survey included data on 8444 female respondents, the 2012 survey included data on 7954 female respondents, and the 2014 survey included data on 31 079 female respondents.

### PEPFAR Funding for PMTCT

Between 2004 and 2006, all financial data were redacted from the COPs. Overall, we assigned 53% of total planned PEPFAR expenditures for PMTCT to specific provinces; the remainder could not be assigned to specific provinces either because they had been allocated to nationwide programs (16%) or implementing partners working across province borders (10%) or because there was insufficient information to assign funding to a specific implementing partner (20%) (percentages total 99% owing to rounding; see eFigure 1 in the Supplement).

### Neonatal Mortality

Our analysis included 33 181 neonates (16 311 girls and 16 870 boys) born 1 to 60 months before the survey, 822 of whom died during the first month of life. Neonatal mortality was not associated with annual PCF or cumulative PCF (Table 1; eFigure 2 in the Supplement).

**Table 1.** Association Between PEPFAR Funding for PMTCT and Neonatal Mortality in Kenya

Variable	Annual PCF		Fully Adjusted Cumulative PCF^b^
	Minimally Adjusted^a^	Fully Adjusted^b^	
No lag			
No./total No.	591/23 814	591/23 814	712/29 432
RR (95% CI)			
For $0 to 1 IQR increase^c^	1.17 (0.92-1.49)	1.21 (0.96-1.52)	0.98 (0.83-1.17)
For $0 to maximum increase^d^	1.56 (0.79-3.06)	1.71 (0.90-3.27)	0.90 (0.29-2.78)
*P* value	.22	.12	.85
1-y Lag			
No./total No.	630/24 975	630/24 975	711/29 386
RR (95% CI)			
For $0 to 1 IQR increase^c^	1.03 (0.83-1.28)	1.05 (0.84-1.31)	1.03 (0.86-1.23)
For $0 to maximum increase^d^	1.08 (0.59-1.99)	1.15 (0.62-2.14)	1.18 (0.36-3.80)
*P* value	.80	.66	.79
2-y Lag			
No./total No.	623/25 904	623/25 904	696/29 067
RR (95% CI)			
For $0 to 1 IQR increase^c^	0.99 (0.82-1.20)	1.00 (0.83-1.21)	1.11 (0.92-1.34)
For $0 to maximum increase^d^	0.98 (0.57-1.68)	1.01 (0.60-1.70)	2.04 (0.60-6.96)
*P* value	.94	.98	.26
3-y Lag			
No./total No.	646/25 763	646/25 763	693/28 313
RR (95% CI)			
For $0 to 1 IQR increase^c^	0.94 (0.75-1.18)	0.96 (0.77-1.19)	1.06 (0.86-1.31)
For $0 to maximum increase^d^	0.85 (0.45-1.59)	0.88 (0.47-1.65)	1.47 (0.36-5.98)
*P* value	.60	.69	.59

Abbreviations: IQR, interquartile range; PCF, per capita funding; PEPFAR, US President’s Emergency Plan for AIDS Relief; PMTCT, prevention of mother-to-child transmission of HIV; RR, risk ratio.

^a^ Adjusted only for province and calendar year.

^b^ Adjusted for province, calendar year, household wealth quintile, water and sanitation access, urban vs rural status, mosquito net ownership, maternal age at birth, educational level, ethnicity, religion, marital status, exposure to mass media, child’s sex, short interval preceding birth, and birth order.

^c^ A $0 to 1 IQR increase in PEPFAR funding for PMTCT corresponds to a $0.33 change in annual PCF and a $0.83 change in cumulative PCF.

^d^ A $0 to maximum increase in PEPFAR funding for PMTCT corresponds to a $0.93 change in annual PCF and a $5.46 change in cumulative PCF.

### Infant Mortality

**Figure 1.**
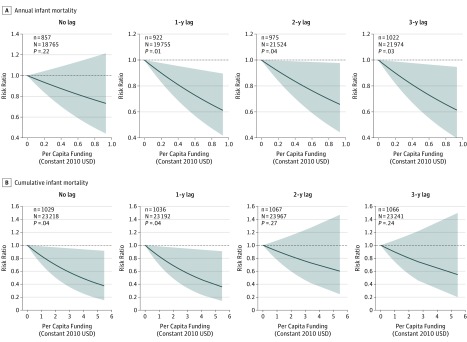
Relative Risk for the Association Between Annual and Cumulative Per Capita US President’s Emergency Plan for AIDS Relief Funding for Prevention of Mother-to-Child Transmission of HIV and Infant Mortality Each panel presents the number of deaths (n) out of the number of infants with complete exposure data (N). Shaded areas indicate 95% CIs. Estimates are adjusted for province, year of birth, household wealth quintile, water and sanitation access, urban vs rural status, mosquito net ownership, maternal age at birth, educational level, ethnicity, religion, marital status, exposure to mass media, child's sex, birth order, and short interval preceding birth. USD indicates US dollars.

The 2003, 2008-2009, and 2014 surveys included birth histories for 128 199 children (13 197 girls and 13 679 boys), 26 876 of whom were born 12 to 60 months before the survey. Of these, 1222 died within the first year of life. After a 1-year lag, a $0.33 increase in annual PCF, corresponding to the difference between the 75th and 25th percentiles (interquartile range) of observed annual PCF data, was associated with a significant 16% (95% CI, 4%-27%) reduction in infant mortality in the fully adjusted model (Table 2). This reduction was sustained after 2- and 3-year lags (Figure 1), but was not significant in the unlagged model. For cumulative PCF, a $0.83 increase (reflecting the interquartile range of cumulative PCF) was similarly associated with a 14% (95% CI, 1%-25%) decrease in infant mortality in the unlagged model. This reduction was sustained after a 1-year lag, with associations attenuating and becoming nonsignificant after subsequent lags.

**Table 2.** Association Between PEPFAR Funding for PMTCT and Infant Mortality in Kenya

Variable	Annual PCF		Fully Adjusted Cumulative PCF^b^
	Minimally Adjusted^a^	Fully Adjusted^b^	
No lag			
No./total No.	857/18 765	857/18 765	1029/23 218
RR (95% CI)			
For $0 to 1 IQR increase^c^	0.88 (0.73-1.07)	0.90 (0.75-1.07)	0.86 (0.75-0.99)
For $0 to maximum increase^d^	0.70 (0.41-1.20)	0.73 (0.44-1.21)	0.37 (0.15-0.91)
*P* value	.20	.22	.04
1-y Lag			
No./total No.	922/19 755	922/19 755	1036/23 192
RR (95% CI)			
For $0 to 1 IQR increase^c^	0.83 (0.72-0.95)	0.84 (0.73-0.96)	0.86 (0.74-0.99)
For $0 to maximum increase^d^	0.59 (0.40-0.87)	0.61 (0.42-0.90)	0.36 (0.14-0.91)
*P* value	.01	.01	.04
2-y Lag			
No./total No.	975/21 534	975/21 534	1067/23 967
RR (95% CI)			
For $0 to 1 IQR increase^c^	1.19 (0.82-1.74)	0.86 (0.75-0.99)	0.93 (0.81-1.06)
For $0 to maximum increase^d^	0.51 (0.32-0.81)	0.66 (0.44-0.98)	0.60 (0.24-1.47)
*P* value	.26^e^	.04	.27
3-y Lag			
No./total No.	1022/21 974	1022/21 974	1066/23 241
RR (95% CI)			
For $0 to 1 IQR increase^c^	0.83 (0.71-0.97)	0.84 (0.72-0.98)	0.91 (0.78-1.06)
For $0 to maximum increase^d^	0.60 (0.39-0.92)	0.61 (0.40-0.95)	0.55 (0.20-1.50)
*P* value	.02	.03	.24

Abbreviations: IQR, interquartile range; PCF, per capita funding; PEPFAR, US President’s Emergency Plan for AIDS Relief; PMTCT, prevention of mother-to-child transmission of HIV; RR, risk ratio.

^a^ Adjusted only for province and calendar year.

^b^ Adjusted for province, calendar year, household wealth quintile, water and sanitation access, urban vs rural status, mosquito net ownership, maternal age at birth, educational level, ethnicity, religion, marital status, exposure to mass media, child’s sex, short interval preceding birth, and birth order.

^c^ A $0 to 1 IQR increase in PEPFAR funding for PMTCT corresponds to a $0.33 change in annual PCF and a $0.83 change in cumulative PCF.

^d^ A $0 to maximum increase in PEPFAR funding for PMTCT corresponds to a $0.93 change in annual PCF and a $5.46 change in cumulative PCF.

^e^ A significant departure from linearity was detected; the *P* value reflects the significance of the nonlinear association.

### HIV Testing During ANC

**Figure 2.**
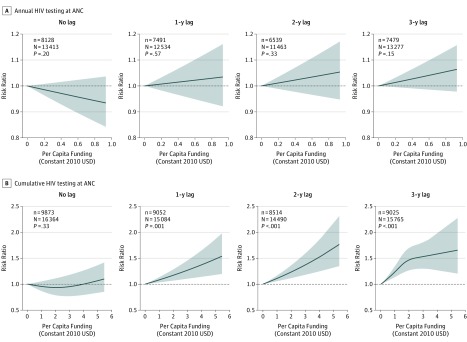
Relative Risk for the Association Between Annual and Cumulative Per Capita US President’s Emergency Plan for AIDS Relief Funding for Prevention of Mother-to-Child Transmission of HIV and HIV Testing During Antenatal Care (ANC) HIV testing during ANC is defined as receiving counseling on prevention of mother-to-child transmission, undergoing testing for HIV, and receiving the results of this HIV test during ANC. Each panel presents the number of women tested (n) out of the number of women reporting a recent birth with complete exposure data (N). Shaded areas indicate 95% CIs. Estimates are adjusted for province, year of birth, household wealth quintile, water and sanitation access, urban vs rural status, mosquito net ownership, maternal age at last birth, educational level, ethnicity, religion, marital status, parity, and exposure to mass media. USD indicates US dollars.

The 2007, 2008-2009, 2012, and 2014 surveys included data on 57 721 female respondents (mean [SD] age, 28.0 [6.7] years), 21 488 of whom had recently given birth. Of the 20 775 women with data on HIV testing, 11 984 (57.7%) received HIV testing during ANC. Annual PCF was not associated with HIV testing during ANC in any model, although there were associations with cumulative PCF (Table 3). After a 1-year lag, a $0.83 increase in cumulative PCF was associated with a 7% (95% CI, 3%-11%) increase in HIV testing during ANC. This association intensified with subsequent lags: a $0.83 increase in cumulative PCF was associated with a 9% (95% CI, 5%-14%) increase after a 2-year lag and a 19% (95% CI, 11%-28%) increase after a 3-year lag (Figure 2).

**Table 3.** Association Between PEPFAR Funding for PMTCT and HIV Testing During Antenatal Care in Kenya

Variable	Annual PCF		Fully Adjusted Cumulative PCF^b^
	Minimally Adjusted^a^	Fully Adjusted^b^	
No lag			
No./total No.	8128/13 413	8128/13 413	9873/16 364
RR (95% CI)			
For $0 to 1 IQR increase^c^	0.99 (0.96-1.03)	0.98 (0.94-1.01)	0.96 (0.89-1.04)
For $0 to maximum increase^d^	0.97 (0.88-1.08)	0.93 (0.84-1.04)	1.10 (0.85-1.42)
*P* value	.61	.20	.33
1-y Lag			
No./total No.	7491/12 534	7491/12 534	9052/15 084
RR (95% CI)			
For $0 to 1 IQR increase^c^	1.01 (0.97-1.06)	1.01 (0.97-1.05)	1.07 (1.03-1.11)
For $0 to maximum increase^d^	1.04 (0.93-1.16)	1.03 (0.92-1.16)	1.54 (1.20-1.98)
*P* value	.48	.57	<.001
2-y Lag			
No./total No.	6539/11 463	6539/11 463	8514/14 490
RR (95% CI)			
For $0 to 1 IQR increase^c^	1.01 (0.97-1.04)	1.02 (0.98-1.06)	1.09 (1.05-1.14)
For $0 to maximum increase^d^	1.02 (0.93-1.12)	1.05 (0.95-1.17)	1.76 (1.35-2.31)
*P* value	.67	.33	<.001
3-y Lag			
No./total No.	7479/13 277	7479/13 277	9025/15 765
RR (95% CI)			
For $0 to 1 IQR increase^c^	1.00 (0.97-1.03)	1.02 (0.99-1.05)	1.19 (1.11-1.28)
For $0 to maximum increase^d^	1.01 (0.93-1.09)	1.06 (0.98-1.16)	1.65 (1.20-2.28)
*P* value	.88	.15	<.001^e^

Abbreviations: IQR, interquartile range; PCF, per capita funding; PEPFAR, US President’s Emergency Plan for AIDS Relief; PMTCT, prevention of mother-to-child transmission of HIV; RR, risk ratio.

^a^ Adjusted only for province and calendar year.

^b^ Adjusted for province, calendar year, household wealth quintile, water and sanitation access, urban vs rural status, mosquito net ownership, maternal age at last birth, educational level, ethnicity, religion, marital status, parity, and exposure to mass media.

^c^ A $0 to 1 IQR increase in PEPFAR funding for PMTCT corresponds to a $0.33 change in annual PCF and a $0.83 change in cumulative PCF.

^d^ A $0 to maximum increase in PEPFAR funding for PMTCT corresponds to a $0.93 change in annual PCF and a $5.46 change in cumulative PCF.

^e^ A significant departure from linearity was detected; the *P* value reflects the significance of the nonlinear association.

### Estimation of Potential Lives Saved

Based on the 1-year lagged model, increasing PEPFAR funding for PMTCT from $0 to $0.33 in annual PCF may be associated with an estimated reduction of 118 039 infant deaths between 2004 and 2014, and increasing funding from $0 to $0.93 in annual PCF may be associated with an estimated reduction of 286 438 infant deaths during the same period. These estimates are conservative and do not include benefits accrued among mothers or among children older than 1 year. When data from KAIS 2012 were included, the estimated number of lives saved was appreciably larger: a $0.33 increase in annual PCF was associated with a 36% (95% CI, 12%-53%) reduction in infant mortality after a 1-year lag, which, during 2004-2014, may be associated with 273 924 fewer infant deaths at funding levels of $0.33 in annual PCF and 547 179 fewer infant deaths at funding levels of $0.93.

### Sensitivity Analyses

Sensitivity analyses for our unlagged, 1-year lagged, and 3-year lagged models generally corresponded with the main results: for infant mortality, annual PCF remained associated with a 13% to 19% reduction when province-years with missing data were assigned $0.04 to $0.32 in annual PCF or with inverse probability weighting (eTables 3-5 in the Supplement). When we investigated the association between PEPFAR funding and infant mortality by maternal HIV status, PEPFAR was significantly associated with lower rates of mortality among infants born to HIV-negative mothers in the unlagged and 1-year lagged model but not among infants born to HIV-positive mothers (eTable 6 in the Supplement). However, owing to the small number of deaths among infants with known maternal HIV status (214 deaths among the 3803 infants born to HIV-negative mothers and 45 deaths among the 369 infants born to HIV-positive mothers), there was no evidence that PEPFAR funding had significantly different associations in the 2 groups.

## Discussion

Our study joins a growing body of literature that suggests that PEPFAR has benefited population health.^12,13,14,27,28,29^ Using publicly available data, we found evidence that PEPFAR funding for PMTCT is associated with reduced infant mortality and increased HIV testing during ANC in Kenya. Our analytic approach provides stronger evidence for the benefits of PEPFAR funding than do other designs, such as pre-post designs that fail to account for secular trends, which have been used previously to assess PEPFAR’s effectiveness.^30^

Earlier studies of PEPFAR’s effectiveness focused on a shorter period (2004-2010) and had not found significantly greater reductions in infant mortality among PEPFAR-focus countries relative to nonfocus countries.^14,15^ However, a recent multicountry ecologic study investigating the effect of PEPFAR funding during 2004-2014 reported significant associations between PEPFAR-focus country status and reduced child mortality.^31^ Our significant findings for infant mortality similarly may stem from the fact that our study period (2004-2014) was longer than for most previous studies and may have been better able to investigate the outcome of funding over a longer period in the past than was possible in previous studies. Our significant findings may also stem from 1 or more other factors: funding for PMTCT may be more directly associated with infant mortality than overall PEPFAR funding; our study was not confounded by country-level factors; and Kenya received more PEPFAR funding than most other PEFPAR countries and may have experienced correspondingly greater benefits, providing greater statistical power to detect associations than in previous studies. Despite its association with improvements in infant mortality and HIV testing during ANC, PEPFAR funding was not associated with neonatal mortality, perhaps because neonatal mortality may be more resistant than postneonatal mortality to public health interventions.^9^

### Delayed Associations With Annual Funding

Annual funding was not associated with reduced infant mortality in the year of allocation but became beneficial at later lags (Figure 1). Although not significant, similar trajectories were observed for HIV testing during ANC and neonatal mortality, suggesting that the full outcome of annual funding may not be observable for several years across many outcomes. This delay may reflect logistical delays as PEPFAR funds are absorbed by local PMTCT programs but may also reflect biological realities. There is a 9-month lag between conception and birth and another 12-month lag between birth and ascertaining infant survival. Both logistic and biological factors should be considered when defining a program evaluation’s time horizon. Our finding that annual funding is significantly associated with infant mortality, but not with HIV testing during ANC, may suggest that other PMTCT-related activities, such as provision of antiretroviral therapy to HIV-positive mothers and exposed infants, are more proximally associated with reduced child mortality. However, because cumulative funding was positively associated with increased HIV testing during ANC, it is also possible that sustained investments in PMTCT programs, rather than high levels of annual funding, are necessary to result in increased access to HIV testing during ANC.

### Cumulative Funding and Threshold Effects

When using cumulative funding rather than annual funding, we observed more significant associations after shorter lags. Compared with annual funding, cumulative funding may better reflect sustained investments in infrastructure and personnel. The 2- and 3-year lagged cumulative PCFs were not significantly associated with infant mortality; however, these estimates should be interpreted with caution, as many of the 95% CIs for these associations between PEPFAR funding levels and infant mortality were wide and the point estimates still suggest a strong association between funding and reduced mortality. Alternatively, the association of cumulative funding with infant mortality may fade over time. The significant nonlinear association between 3-year lagged cumulative funding and HIV testing during ANC may suggest a threshold effect for cumulative PCF. However, in our data extracted from the Country Operational Reports, as of 2014 only 1 of 8 provinces (Nyanza) had received more than $2.68 in cumulative PCF; this apparent threshold may be driven largely by a single province. The joint findings of the annual PCF and cumulative PCF models suggest that there is a delayed association of annual funding with PMTCT-related outcomes, that the association of cumulative funding is greater than the association of spending in any individual year, and that there is no evidence of diminishing returns to PEPFAR’s investment in PMTCT programs.

### Robustness of Results

Our findings were relatively robust to sensitivity analyses assessing possible bias owing to missing funding data. Only under unlikely scenarios where missing province-years received very high annual PCF ($0.56-$0.93) did our results for infant mortality become attenuated across all lags. Discrepancies between the main analysis and sensitivity analyses either reflected the detection of new nonlinear associations or they occurred under unlikely scenarios in which missing province-years received very high annual PCF. These analyses suggest that, despite limitations of COPs as a source of regional funding data, our findings are unlikely to be explained by missing data patterns.

When we examined whether the association between PEPFAR funding and infant mortality differed by mothers’ HIV status, PEPFAR was not associated with significantly lower infant mortality among infants of HIV-positive mothers; however, this analysis was limited by the small sample of mothers with known HIV status and because HIV status was ascertained at the time of the interview, which would have been prior to the child’s birth, and could have induced misclassification of women’s HIV status during pregnancy. In contrast, infants of HIV-negative mothers experienced reduced mortality, possibly reflecting positive spillover effects including improved health literacy, health professional training, and health system resources.

### Public Health Relevance

This study illustrates how cost-effective, large-scale program evaluations can be conducted using preexisting data sources. We relied exclusively on COPs, which are produced annually by all PEPFAR focus countries, and the Demographic and Health Survey and the AIDS Indicator Survey. At least 1 Demographic and Health Survey or AIDS Indicator Survey has been conducted in all 31 countries producing COPs. Consequently, our approach may be applied in other geographical settings and adapted for other HIV-related health outcomes. PEPFAR and other international donors seeking to evaluate programmatic outcomes might consider routinely collecting annual data on financial expenditures disaggregated by geography. Linking these financial data to the Demographic and Health Survey, AIDS Indicator Survey, or ongoing PEPFAR-funded population-based HIV impact assessment surveys would enable future evaluations of outcomes. Recently released subnational-level data on PEPFAR’s programmatic activities for 2015-2016^4^ could also be used in future evaluations.

Investigating the association between the amount of funding and health outcomes addresses policy questions about the effectiveness of programs and can inform allocation of future funds. Our study follows 3 previous studies investigating the amount of PEPFAR funding as an exposure and resulting associations with related health and behavioral outcomes, with varying findings.^27,28,33^ For example, Lo and colleagues^33^ did not observe an association between funding for abstinence and being faithful activities, which are designed to delay age of first sexual activity and reduce individuals’ number of sexual partners, and reduced high-risk sexual behavior. Access to a range of literature on the associations between funding for specific HIV prevention approaches and population-level health improvements can help policy makers decide which evidence-informed activities to fund and increase allocation of funds to approaches likely to be effective.

### Limitations

This study has limitations. As with many observational studies, our analysis is vulnerable to bias owing to residual confounding and missing exposure data, making the establishment of causality difficult. We sought to minimize unmeasured confounding by adjusting for fixed effects of province, which controls for unobserved time-invariant province characteristics, and for year, which controls for nationwide secular trends. However, our analysis is vulnerable to confounding by province characteristics that both vary over time and are correlated with PEPFAR funding for PMTCT, such as investment in antimalarial campaigns or child health programing, both of which increased drastically during the early 2000s.^2,32^ We strove to partially capture time-varying province characteristics by adjusting for individual variables, such as mosquito net ownership. However, bias from time-varying confounding is possible, particularly if PEPFAR preferentially allocated funds to implementing partners working in provinces with improving health outcomes. As evidenced by their relatively wide 95% CIs, there is still substantial uncertainty around many of our point estimates, and we may not have had sufficient power to detect all important associations. In addition, the data source for our exposure, COPs, describes annual planned expenditures, which may not reflect actual expenditures, and disaggregate funding by implementing partner rather than by geography, limiting our ability to assign funding to provinces. We expect these administrative processes to result in random measurement error and underestimate associations. However, systematic bias could occur if, for example, implementing partners working in provinces with strong health systems reported higher-quality information in COPs than those working in provinces with weak health systems. Furthermore, our analysis links province-level funding to respondent-level outcomes and does not account for variation in the distribution of PEPFAR-funded activities within a province or variation in individuals’ engagement with PEPFAR-funded PMTCT programs. Thus, these findings are best interpreted as the benefits associated with living in a province receiving a given level of PEPFAR funding and likely underestimate the benefits of interacting directly with a PEPFAR-funded PMTCT program. Similarly, because health outcome data were unavailable after 2014, our analysis does not capture the outcome of recent PEPFAR-funded PMTCT-related activities. Finally, although we investigated multiple definitions of the funding measures, we did not formally adjust for multiple comparisons. Although not all our findings reach statistical significance, the point estimates for the association of funding with both infant mortality and HIV testing in ANC generally provide a consistent picture from a mechanistic point of view, which helps to strengthen confidence in our findings. By presenting our results for these analyses in graphical and tabular form, we hope to allow readers to consider the totality of the evidence when interpreting our results.

## Conclusions

Funding by PEPFAR for PMTCT was associated with reduced infant mortality and increased HIV testing during ANC in Kenya. This research illustrates how preexisting data sources can be used to conduct cost-effective, large-scale program evaluations in a robust and timely manner.

## Notes

Supplement. **eAppendix.** Methods **eFigure 1.** Assigning PEPFAR funding to Kenyan provinces using Country Operational Plans (COPs) **eTable 1.** Five-Year Infant and Neonatal Mortality by Survey **eTable 2.** Exemplary Calculations for Lagged Annual and Cumulative Per-Capita Funding **eFigure 2.** Association Between Annual (a) and Cumulative (b) Per Capita PEPFAR Funding for PMTCT and Neonatal Mortality **eTable 3.** Summary of Sensitivity Analyses Results for Infant Mortality **eTable 4.** Summary of Sensitivity Analyses Results for HIV Testing at ANC **eTable 5.** Summary of Sensitivity Analyses for Neonatal Mortality **eTable 6.** Association Between Dichotomized Pre- vs Post-PEPFAR Funding for PMTCT and Infant Mortality Stratified by Maternal HIV Status **eReferences.**

## References

[1] Measure Evaluation Hill K, Cheluget B, Curtix S, Bicego G, Mahy M. HIV and increases in childhood mortality in Kenya in the late 1980 s to the mid-1990s. https://www.measureevaluation.org/resources/publications/sr-04-26. Accessed March 24, 2017.

[2] Wafula SW, Ikamari LD, K’Oyugi BO In search for an explanation to the upsurge in infant mortality in Kenya during the 1988-2003 period. BMC Public Health. 2012;12:. doi:10.1186/1471-2458-12-441 22708542 PMC3444397

[3] Kenya National AIDS and STI Control Programme (NASCOP) Kenya AIDS Indicator Survey 2012: Final Report. Nairobi, Kenya: NASCOP; 2014.

[4] Office of US Global AIDS Coordinator. PEPFAR Panorama Spotlight. https://data.pepfar.gov/dashboards. Accessed November 25, 2016.

[5] Kenya National Bureau of Statistics, Kenya Ministry of Health, Kenya National AIDS Control Council, Kenya Medical Research Institute, Kenya National Council for Population Development Kenya Demographic and Health Survey 2014: Final Report. Rockville, MD: Kenya National Bureau of Statistics, Ministry of Health, National AIDS Control Council, Kenya Medical Research Institute, National Council for Population Development, DHS Program, ICF International; 2015.

[6] Luzuriaga K, Mofenson LM Challenges in the elimination of pediatric HIV-1 infection. N Engl J Med. 2016;374(8):761-. doi:10.1056/NEJMra1505256 26933850

[7] De Cock KM, Fowler MG, Mercier E, Prevention of mother-to-child HIV transmission in resource-poor countries: translating research into policy and practice. JAMA. 2000;283(9):1175-1182. doi:10.1001/jama.283.9.1175 10703780

[8] Newell ML, Coovadia H, Cortina-Borja M, Rollins N, Gaillard P, Dabis F; Ghent International AIDS Society (IAS) Working Group on HIV Infection in Women and Children Mortality of infected and uninfected infants born to HIV-infected mothers in Africa: a pooled analysis. Lancet. 2004;364(9441):1236-1243. doi:10.1016/S0140-6736(04)17140-7 15464184

[9] Liu L, Oza S, Hogan D, Global, regional, and national causes of child mortality in 2000-13, with projections to inform post-2015 priorities: an updated systematic analysis. Lancet. 2015;385(9966):430-440. doi:10.1016/S0140-6736(14)61698-6 25280870

[10] Luboga SA, Stover B, Lim TW, Did PEPFAR investments result in health system strengthening? a retrospective longitudinal study measuring non-HIV health service utilization at the district level. Health Policy Plan. 2016;31(7):897-909. doi:10.1093/heapol/czw009 27017824 PMC4977428

[11] Lee MM, Izama MP Aid externalities: evidence from PEPFAR in Africa. World Dev. 2015;67:281-294. doi:10.1016/j.worlddev.2014.10.001

[12] Bendavid E, Bhattacharya J The President’s Emergency Plan for AIDS Relief in Africa: an evaluation of outcomes. Ann Intern Med. 2009;150(10):688-695. doi:10.7326/0003-4819-150-10-200905190-00117 19349625 PMC2892894

[13] Bendavid E, Holmes CB, Bhattacharya J, Miller G HIV development assistance and adult mortality in Africa. JAMA. 2012;307(19):2060-2067. doi:10.1001/jama.2012.2001 22665105 PMC3434229

[14] Cohen RL, Li Y, Giese R, Mancuso JD An evaluation of the President’s Emergency Plan for AIDS Relief effect on health systems strengthening in sub-Saharan Africa. J Acquir Immune Defic Syndr. 2013;62(4):471-479. doi:10.1097/QAI.0b013e3182816a86 23254150

[15] Duber HC, Coates TJ, Szekeras G, Kaji AH, Lewis RJ Is there an association between PEPFAR funding and improvement in national health indicators in Africa? a retrospective study. J Int AIDS Soc. 2010;13:21. doi:10.1186/1758-2652-13-21 20540795 PMC2895577

[16] Central Bureau of Statistics–Kenya Kenya Demographic and Health Survey 2003. Calverton, MD: Central Bureau of Statistics, Ministry of Health, and ORC Macro; 2004.

[17] Kenya National AIDS and STI Control Programme (NASCOP) Kenya AIDS Indicator Survey 2007: Final Report. Nairobi, Kenya: NASCOP; 2009.

[18] Kenya National Bureau of Statistics, National AIDS Control Council/Kenya, National AIDS/STD Control Programme/Kenya, Ministry of Public Health and Sanitation/Kenya, Kenya Medical Research Institute. Kenya Demographic and Health Survey 2008-09. Calverton, MD: KNBS and ICF Macro; 2010.

[19] Hallett TB, Gregson S, Kurwa F, Measuring and correcting biased child mortality statistics in countries with generalized epidemics of HIV infection. Bull World Health Organ. 2010;88(10):761-768. doi:10.2471/BLT.09.071779 20931061 PMC2947040

[20] Zaba B, Marston M, Floyd S The Effect of HIV on Child Mortality Trends in Sub-Saharan Africa. New York, NY: United Nations Population Division, Department of Economic and Social Affairs; 2003.

[21] United States President’s Emergency Plan for AIDS Relief County operational plans. https://www.pepfar.gov/countries/cop/index.htm. Accessed November 25, 2016.

[22] Habicht JP, Victora CG, Vaughan JP Evaluation designs for adequacy, plausibility and probability of public health programme performance and impact. Int J Epidemiol. 1999;28(1):10-18. doi:10.1093/ije/28.1.10 10195658

[23] World Bank Subnational population database. https://data.worldbank.org/data-catalog/subnational-population. Accessed October 5, 2017.

[24] Fitzmaurice G, Laird N, Ware J Applied Longitudinal Analysis. 2nd ed Hoboken, NJ: John Wiley & Sons Inc; 2011. doi:10.1002/9781119513469

[25] Durrleman S, Simon R Flexible regression models with cubic splines. Stat Med. 1989;8(5):551-561. doi:10.1002/sim.4780080504 2657958

[26] Seaman SR, White IR Review of inverse probability weighting for dealing with missing data. Stat Methods Med Res. 2013;22(3):278-295. doi:10.1177/0962280210395740 21220355

[27] Wagner Z, Barofsky J, Sood N PEPFAR funding associated with an increase in employment among males in ten sub-Saharan African countries. Health Aff (Millwood). 2015;34(6):946-953. doi:10.1377/hlthaff.2014.1006 26056199 PMC4782769

[28] Chin RJ, Sangmanee D, Piergallini L PEPFAR funding and reduction in HIV infection rates in 12 focus sub-Saharan African countries: a quantitative analysis. Int J MCH AIDS. 2015;3(2):150-158. 27621994 PMC5005989

[29] Lima VD, Granich R, Phillips P, Williams B, Montaner JS Potential impact of the US President’s Emergency Plan for AIDS relief on the tuberculosis/HIV coepidemic in selected sub-Saharan African countries. J Infect Dis. 2013;208(12):2075-2084. doi:10.1093/infdis/jit406 23911712 PMC3836466

[30] Office of the US Global AIDS Coordinator and Health Diplomacy, US State Department PEPFAR: 2017 annual report to Congress. https://www.pepfar.gov/documents/organization/267809.pdf. Accessed February 13, 2018.

[31] Kim Y The effectiveness of PEPFAR’s funding for women and children with HIV/AIDS. Int J Health Plann Manage. 2019;34(1):e896-e916. doi:10.1002/hpm.2706 30451315

[32] Keats EC, Macharia W, Singh NS, Accelerating Kenya’s progress to 2030: understanding the determinants of under-five mortality from 1990 to 2015. BMJ Glob Health. 2018;3(3):e000655. doi:10.1136/bmjgh-2017-000655 29862055 PMC5969726

[33] Lo NC, Lowe A, Bendavid E Abstinence funding was not associated with reductions in HIV risk behavior in sub-Saharan Africa. Health Aff (Millwood). 2016;35(5):856-863. doi:10.1377/hlthaff.2015.0828 27140992

