# Supplementary material for: Association of the US President’s Emergency Plan for AIDS Relief’s Funding With Prevention of Mother-to-Child Transmission of HIV in Kenya
Source: JAMA Netw Open. 2019 Sep 13;2(9):e1911318. doi: 10.1001/jamanetworkopen.2019.11318 (PMC6745052; doi:10.1001/jamanetworkopen.2019.11318)
Supplement: Supplement. — eAppendix. Methods eFigure 1. Assigning PEPFAR funding to Kenyan provinces using Country Operational Plans (COPs) eTable 1. Five-Year Infant and Neonatal Mortality by Survey eTable 2. Exemplary Calculations for Lagged Annual and Cumulative Per-Capita Funding eFigure 2. Association Between Annual (a) and Cumulative (b) Per Capita PEPFAR Funding for PMTCT and Neonatal Mortality eTable 3. Summary of Sensitivity Analyses Results for Infant Mortality eTable 4. Summary of Sensitivity Analyses Results for HIV Testing at ANC eTable 5. Summary of Sensitivity Analyses for Neonatal Mortality eTable 6. Association Between Dichotomized Pre- vs Post-PEPFAR Funding for PMTCT and Infant Mortality Stratified by Maternal HIV Status eReferences. [file jamanetwopen-2-e1911318-s001.pdf]

Supplementary Online Content

Barnhart DA, Tsikhutsu I, Kirui D, et al. Association of the US President's Emergency Plan for AIDS Relief's funding with prevention of mother-to-child transmission of HIV in Kenya. *JAMA Netw Open.* 2019;2(9):e1911318. doi:10.1001/jamanetworkopen.2019.11318

eAppendix. Methods
eFigure 1. Assigning PEPFAR funding to Kenyan provinces using Country Operational Plans (COPs)
eTable 1. Five-Year Infant and Neonatal Mortality by Survey
eTable 2. Exemplary Calculations for Lagged Annual and Cumulative Per-Capita Funding
eFigure 2. Association Between Annual (a) and Cumulative (b) Per Capita PEPFAR Funding for PMTCT and Neonatal Mortality
eTable 3. Summary of Sensitivity Analyses Results for Infant Mortality
eTable 4. Summary of Sensitivity Analyses Results for HIV Testing at ANC
eTable 5. Summary of Sensitivity Analyses for Neonatal Mortality
eTable 6. Association Between Dichotomized Pre- vs Post-PEPFAR Funding for PMTCT and Infant Mortality Stratified by Maternal HIV Status
eReferences.

This supplementary material has been provided by the authors to give readers additional information about their work.

© 2019 Barnhart DA et al. *JAMA Network Open.*

eAppendix. Methods

A. Birth history data from KAIS 2012

Although the KAIS 2012 gathered data on birth histories, we excluded it in our primary analysis of neonatal and infant mortality. This decision was driven by several factors. First, the KAIS 2012 did not report on birth history data in their final report, which may reflect the fact that women's birth histories, which have traditionally been a major focus of Demographic and Health Surveys such as the KDHS, have not traditionally been a major focus of AIDS Indicator Surveys, such as the KAIS. This omission may also reflect underlying concerns about the data quality. As shown in the table below, the five-year neonatal and infant mortality rate calculated using data from the KAIS 2012 was substantially lower than that calculated from other surveys, even when the time frame covered by those other surveys overlapped substantially with the time frame covered by the KAIS 2012.

Due these underlying concerns, we choose to omit KAIS 2012 from our primary analysis of infant and neonatal mortality. We do provide secondary results estimating lives saved with KAIS 2012 data included, and it can be seen that the impact is substantial, with a dramatically higher number of lives saved estimated.

B. Data Extraction from Country Operational Plans (COPs)

Each year, PEPFAR focus countries submit country operational plans (COPs) containing PEPFAR's annual planned expenditures disaggregated by implementation partner and budget code. We identified annual PMTCT funding from the COPs using the "MTCT" budget code. Narrative information from the COPs were used to assign dollar amounts to one of Kenya's eight provinces. Dollar amounts were converted to 2010 USD using a GDP deflator. We set funding for pre-PEPFAR years to zero.

C. Modeling the risk ratio

We estimated risk ratios and 95% confidence intervals associated with PEPFAR funding for PMTCT using generalized weighted estimating equations (GEEs), using exchangeable working covariance structures with robust standard errors to account for correlations within sampling clusters.[1] To create a representative sample across surveys, we used denormalized weights calculated by multiplying the survey sampling weights by the number of individuals in the survey's sampling frame divided by the survey's sample size.[2] To maximize statistical efficiency, we used the working binomial variance or, if models failed to converge, the working Poisson variance.[3,4] We assessed potential non-linear relationships between funding and health outcomes using restricted cubic splines,[5] selecting spline terms using a SAS macro available on the last author's website[6] and presenting the model with the linear term if non-linear relationships were non-significant. All models adjusted for province and controlled for calendar year using restricted cubic splines. Fully-adjusted models further adjusted for household characteristics, including household wealth quintile, water and sanitation access, urban/rural status, and mosquito net ownership, and respondent characteristics, including education level, ethnicity, religion, marital status, maternal age categorized as <20, 20-34, and ≥35 years of age, and exposure to mass media. For the outcomes of neonatal and infant mortality, fully adjusted models also adjusted for child's sex, short preceding birth interval (≤24 months) and birth order (rather than parity). To account for secular changes in wealth, we developed harmonized wealth quintiles using the first principal component of household assets measured across all surveys.[7]

D. Estimation of potential lives saved

To estimate the number of lives saved by PEPFAR funding for PMTCT, we used the one-year lagged model to predict the probability of infant mortality in each province-year assuming $0, $0.33, and $0.93 in ANN-PCF. We multiplied these predicted probabilities of death by the number of infants born in each province-year between 2004 and 2014 calculated by allocating the UN Population Division's national birth estimates proportionally to the World Bank Group's Subnational Population Database's adult population estimates.

E. Inverse Probability weighting for missing exposure data

For infant mortality, we used inverse probability weighting to adjust for possible bias due to the exclusion of infants with missing data on ANN-PCF.[8] We predicted the probability that each infant was missing data on ANN-PCF

© 2019 Barnhart DA et al. *JAMA Network Open.*

using a logistic regression model that included the outcome, with model covariates and two-way interactions being added to the model when significant at $p<0{\cdot}05$. The inverse of this predicted probability, truncated at the 99th percentile, was used as a weight in the regression of infant mortality on ANN-PCF.

F. Association between PEPFAR funding and infant mortality by maternal HIV status

The sample size for our analysis by maternal HIV status was limited because only the KDHS 2003 and KDHS 2008/2009 gathered both full birth history data and dried blood spots, which were used to assess on maternal HIV status. The sample size for this analysis consisted of 3,803 infants of HIV-negative mothers, 214 of whom had experienced infant mortality, and 369 infants of HIV-positive mothers, 45 of whom had experienced infant mortality. Because financial data from the COPs was redacted between 2004-2006, we were unable to fit models with a continuous exposure in the subset of infants whose mothers' HIV status was known. Instead, we modeled the zero- to three-year lagged association with dichotomized pre- vs. post- PEPFAR funding, adjusted for year and province.

© 2019 Barnhart DA et al. *JAMA Network Open.*

eFigure 1. Assigning PEPFAR funding to Kenyan provinces using Country Operational Plans (COPs)
The number at the top of each bar gives the number of provinces (out of 8) with data on PEPFAR funding for PMTCT in that year.

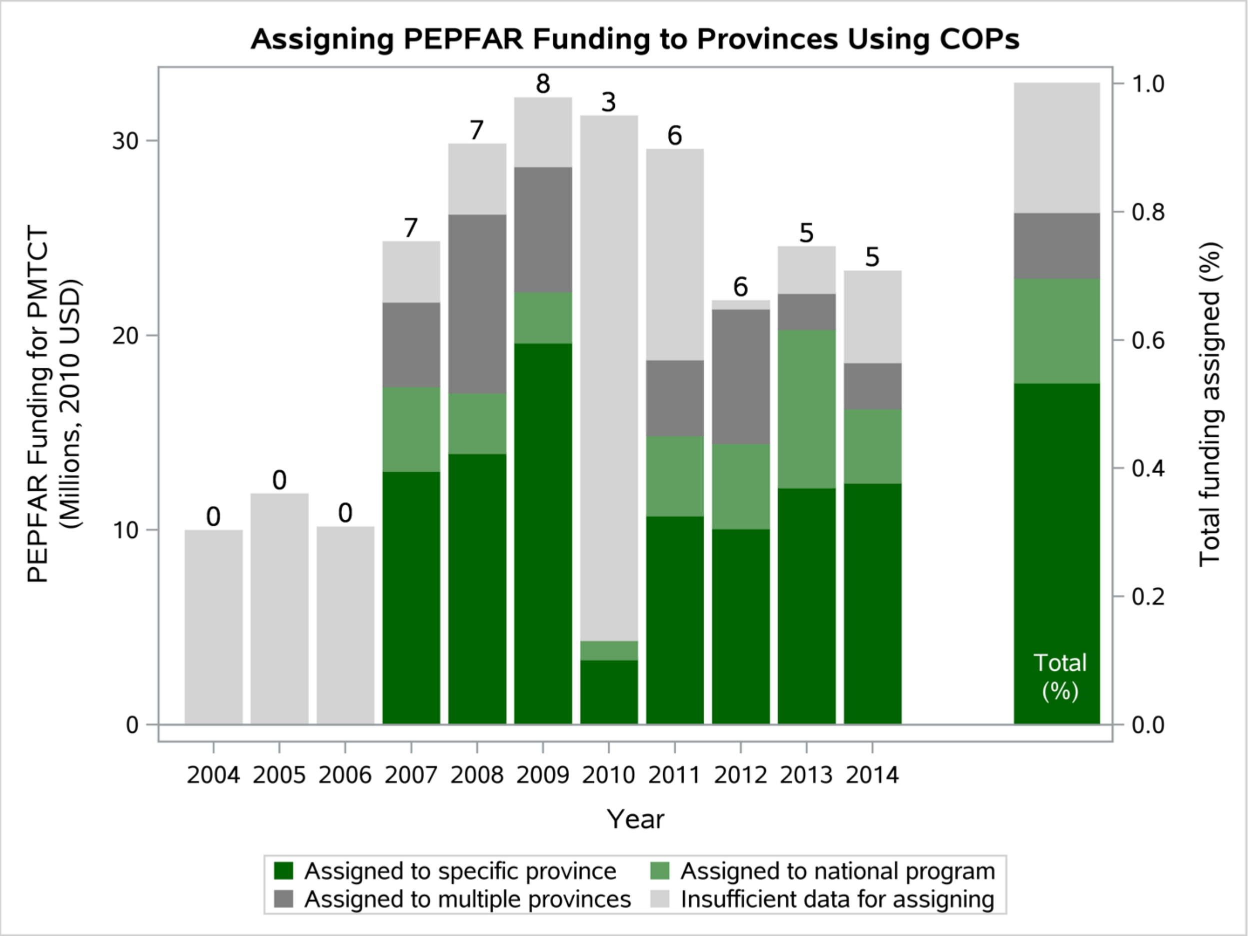

© 2019 Barnhart DA et al. *JAMA Network Open.*

eTable 1. Five-Year Infant and Neonatal Mortality by Survey

		**Infant mortality**			**Neonatal Mortality**		
**Survey**	**Years Covered**	**Deaths**	**Infants**	**Mortality rate % , (95% CI)**	**Deaths**	**Neonates**	**Mortality rate % , (95% CI)**
**KDHS 2003**	1998-2003	349	4750	7.7, (6.7, 8.7)	197	5960	3.3, (2.7, 3.9)
**KDHS 2008**	2003-2009	254	4850	5.4, (4.3, 6.4)	176	6083	3.1, (2.4, 3.8)
**KAIS 2012**	2008-2013	52	3548	2.4, (0, 4.7)	30	4435	0.6, (0.3, 0.8)
**KDHS 2014**	2009-2014	619	17276	3.8, (3.4, 4.2)	449	21138	2.2, (1.9, 2.5)

© 2019 Barnhart DA et al. *JAMA Network Open.*

eTable 2. Exemplary Calculations for Lagged Annual and Cumulative Per-Capita Funding
Calculations are shown for a single province. "·" designates a missing value.

			Annual per-capita funding for PMTCT				Cumulative per-capita funding for PMTCT			
**Year**	**Funding for PMTCT (Millions, 2010 USD)**	**Population (Millions)**	**Unlagged**	**1-year lag**	**2-year lag**	**3-year lag**	**Unlagged**	**1-year lag**	**2-year lag**	**3-year lag**
2000	$0	7·61	$0	$0	$0	$0	$0	$0	$0	$0
2001	$0	7·86	$0	$0	$0	$0	$0	$0	$0	$0
2002	$0	8·12	$0	$0	$0	$0	$0	$0	$0	$0
2003	$0	8·39	$0	$0	$0	$0	$0	$0	$0	$0
2004	·	8·67	·	$0	$0	$0	·	$0	$0	$0
2005	·	8·95	·	·	$0	$0	·	·	$0	$0
2006	·	9·24	·	·	·	$0	·	·	·	$0
2007	$2·57	9·54	$0·28	·	·	·	$0·28	·	·	·
2008	$3·08	9·85	$0·32	$0·28	·	·	$0·60	$0·28	·	·
2009	$5·60	10·17	$0·56	$0·32	$0·28	·	$1·16	$0·60	$0·28	·
2010	$2·03	10·50	$0·19	$0·56	$0·32	$0·28	$1·35	$1·16	$0·60	$0·28
2011	$1·97	10·83	$0·18	$0·19	$0·56	$0·32	$1·53	$1·35	$1·16	$0·60
2012	$0·52	11·17	$0·04	$0·18	$0·19	$0·56	$1·57	$1·53	$1·35	$1·16
2013	$3·02	11·52	$0·25	$0·04	$0·18	$0·19	$1·82	$1·57	$1·53	$1·35
2014	$2·54	11·88	$0·20	$0·25	$0·04	$0·18	$2·02	$1·82	$1·57	$1·53

© 2019 Barnhart DA et al. *JAMA Network Open.*

eFigure 2. Association Between Annual (a) and Cumulative (b) Per Capita PEPFAR Funding for PMTCT and Neonatal Mortality

Each panel presents the number of deaths (n) out of the number of infants with complete exposure data (N). Estimates are adjusted for province; year of birth; household wealth quintile, water and sanitation access, urban/rural status and mosquito net ownership; maternal age at birth, education, ethnicity, religion, marital status, and exposure to mass media; and child's sex, birth order, and short preceding birth interval.

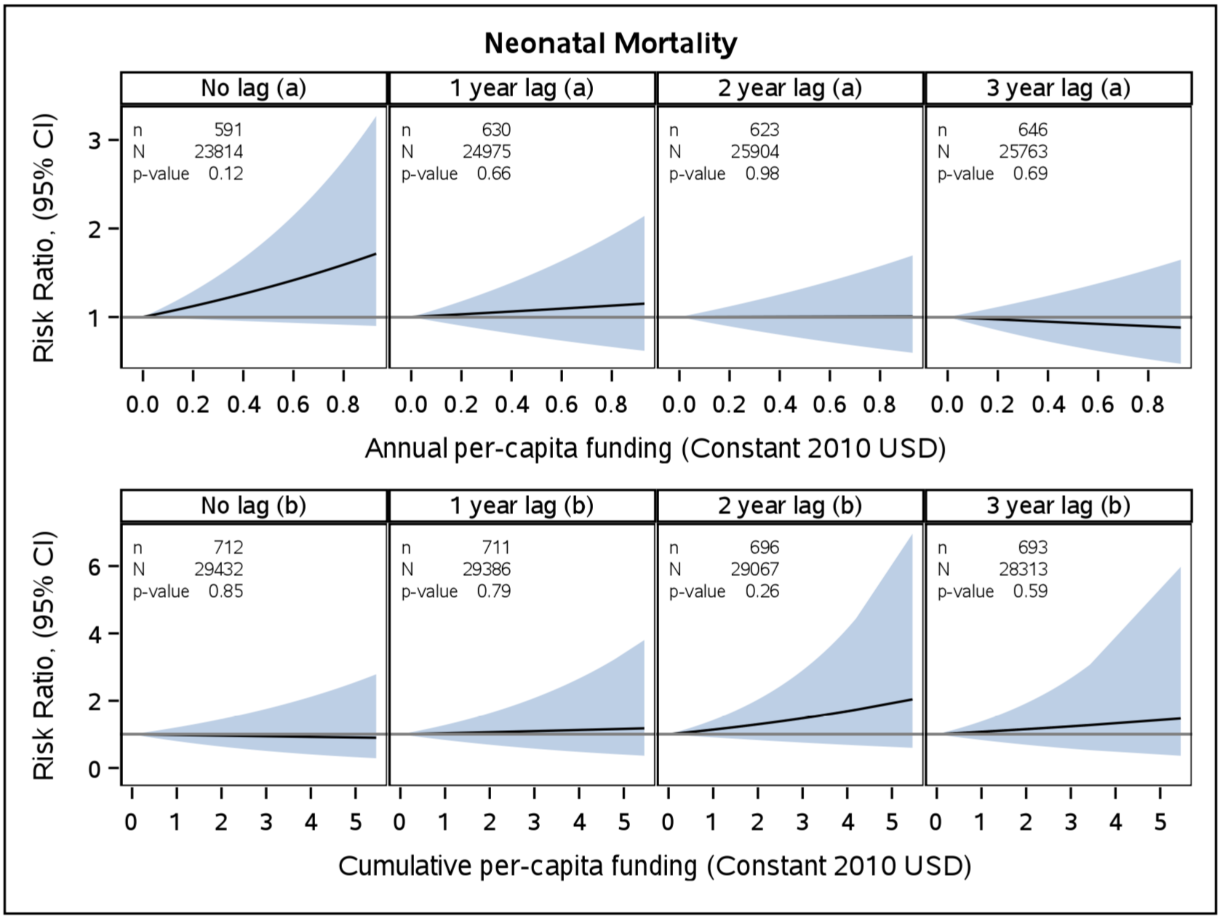

© 2019 Barnhart DA et al. *JAMA Network Open.*

eTable 3. Summary of Sensitivity Analyses Results for Infant Mortality
Cells include the risk ratio (RR) corresponding to a $0·33 increase in annual PEPFAR funding per capita. Table shows p-values for test of linear trend (L) or, if significant departures from linearity were detected, the test for non-linearity (NL) and test for the significance of the curve (C).

	**Infant Mortality**			
	**No lag**	**1 year lag**	**2 year lag**	**3 year lag**
**U**	0.88 (0.73, 1.07)<br>L: 0.20	0.83 (0.72, 0.95)<br>L: 0.01	1.19 (0.82, 1.74)<br>NL: 0.04<br>C: 0.26	0.83 (0.71, 0.97)<br>L: 0.02
**A**	0.90 (0.75, 1.07)<br>L: 0.22	0.84 (0.73, 0.96)<br>L: 0.01	0.86 (0.75, 0.99)<br>L: 0.04	0.84 (0.72, 0.98)<br>L: 0.03
**S1**	0.93 (0.80, 1.08)<br>L: 0.36	0.91 (0.81, 1.02)<br>L: 0.09	1.13 (0.83, 1.52)<br>NL: 0.05<br>C: 0.31	0.87 (0.77, 0.98)<br>L: 0.03
**S2**	0.89 (0.75, 1.06)<br>L: 0.19	0.86 (0.75, 0.98)<br>L: 0.02	1.18 (0.88, 1.59)<br>NL: 0.02<br>C: 0.23	0.85 (0.73, 0.98)<br>L: 0.03
**S3**	0.89 (0.75, 1.05)<br>L: 0.17	0.86 (0.75, 0.99)<br>L: 0.03	1.15 (0.89, 1.49)<br>NL: 0.02<br>C: 0.18	0.85 (0.73, 0.99)<br>L: 0.04
**S4**	0.93 (0.82, 1.05)<br>L: 0.23	0.91 (0.80, 1.03)<br>L: 0.12	1.27 (1.01, 1.59)<br>NL: 0.003<br>C: 0.03	1.01 (0.83, 1.23)<br>NL: 0.05<br>C: 0.95
**S5**	0.96 (0.89, 1.04)<br>L: 0.36	0.95 (0.88, 1.04)<br>L: 0.25	1.02 (0.94, 1.11)<br>L: 0.69	0.96 (0.87, 1.06)<br>L: 0.38
**S6**	0.90 (0.76, 1.07)<br>L: 0.23	0.84 (0.74, 0.96)<br>L: 0.02	1.19 (0.83, 1.69)<br>NL: 0.03<br>C: 0.24	0.81 (0.68, 0.95)<br>L: 0.01

KEY

U=Minimally adjusted analysis
A=Fully adjusted analysis
S1=Missing province-years received $0·04 in annual per capita funding
S2=Missing province-years received $0·23 in annual per capita funding
S3=Missing province-years received $0·32 in annual per capita funding
S4=Missing province-years received $0·56 in annual per capita funding
S5=Missing province-years received $0·93 in annual per capita funding
S6=Inverse probability weighting
■ Funding associated with improved health outcomes
■ Funding significantly associated with improved health outcomes
■ Concave relationship between funding & health outcomes
■ Significant concave relationship between funding & health outcomes
■ Funding associated with worse health outcomes
■ Funding significantly associated with worse health outcomes
☐ Funding not associated with health outcomes

© 2019 Barnhart DA et al. *JAMA Network Open.*

eTable 4. Summary of Sensitivity Analyses Results for HIV Testing at ANC
Cells include the risk ratio (RR) corresponding to a $0·33 increase in annual PEPFAR funding per capita. Table shows p-values for test of linear trend (L) or, if significant departures from linearity were detected, the test for non-linearity (NL) and test for the significance of the curve (C).

	HIV testing at ANC			
	**No lag**	**1 year lag**	**2 year lag**	**3 year lag**
**U**	0.99 (0.96, 1.03)<br>L: 0.61	1.01 (0.97, 1.06)<br>L: 0.48	1.01 (0.97, 1.04)<br>L: 0.67	1.00 (0.97, 1.03)<br>L: 0.88
**A**	0.98 (0.94, 1.01)<br>L: 0.20	1.01 (0.97, 1.05)<br>L: 0.57	1.02 (0.98, 1.06)<br>L: 0.33	1.02 (0.99, 1.05)<br>L: 0.15
**S1**	0.99 (0.97, 1.02)<br>L: 0.61	0.99 (0.96, 1.02)<br>L: 0.44	0.93 (0.88, 0.99)<br>NL: 0.009<br>C: 0.02	1.01 (0.99, 1.04)<br>L: 0.26
**S2**	0.94 (0.88, 1.00)<br>NL: 0.04<br>C: 0.04	1.00 (0.96, 1.03)<br>L: 0.91	1.02 (0.99, 1.05)<br>L: 0.29	1.02 (0.99, 1.05)<br>L: 0.17
**S3**	0.99 (0.95, 1.02)<br>L: 0.43	1.01 (0.97, 1.04)<br>L: 0.78	1.03 (1.00, 1.06)<br>L: 0.08	1.02 (0.99, 1.05)<br>L: 0.18
**S4**	0.99 (0.96, 1.02)<br>L: 0.46	1.02 (0.98, 1.05)<br>L: 0.34	1.04 (1.01, 1.07)<br>L: 0.01	1.01 (0.99, 1.04)<br>L: 0.29
**S5**	0.99 (0.97, 1.02)<br>L: 0.60	1.01 (0.99, 1.03)<br>L: 0.22	1.03 (1.01, 1.04)<br>L: 0.008	1.01 (0.99, 1.03)<br>L: 0.42

KEY

U=Minimally adjusted analysis
A=Fully adjusted analysis
S1=Missing province-years received $0·04 in annual per capita funding
S2=Missing province-years received $0·23 in annual per capita funding
S3=Missing province-years received $0·32 in annual per capita funding
S4=Missing province-years received $0·56 in annual per capita funding
S5=Missing province-years received $0·93 in annual per capita funding
S6=Inverse probability weighting
■ Funding associated with improved health outcomes
■ Funding significantly associated with improved health outcomes
■ Concave relationship between funding & health outcomes
■ Significant concave relationship between funding & health outcomes
■ Funding associated with worse health outcomes
■ Funding significantly associated with worse health outcomes
☐ Funding not associated with health outcomes

© 2019 Barnhart DA et al. *JAMA Network Open.*

eTable 5. Summary of Sensitivity Analyses for Neonatal Mortality
Cells include the risk ratio (RR) corresponding to a $0·33 increase in annual PEPFAR funding per capita. Table show p-values for test of linear trend (L). No significant departures from linearity were detected.

	**Neonatal Mortality**			
	**No lag**	**1 year lag**	**2 year lag**	**3 year lag**
**U**	1.17 (0.92, 1.49)<br>L: 0.22	1.03 (0.83, 1.28)<br>L: 0.80	0.99 (0.82, 1.20)<br>L: 0.94	0.94 (0.75, 1.18)<br>L: 0.60
**A**	1.21 (0.96, 1.52)<br>L: 0.12	1.05 (0.84, 1.31)<br>L: 0.66	1.00 (0.83, 1.21)<br>L: 0.98	0.96 (0.77, 1.19)<br>L: 0.69
**S1**	1.12 (0.92, 1.36)<br>L: 0.28	0.98 (0.81, 1.19)<br>L: 0.86	0.89 (0.75, 1.04)<br>L: 0.14	0.94 (0.80, 1.12)<br>L: 0.50
**S2**	1.13 (0.91, 1.41)<br>L: 0.28	1.02 (0.84, 1.25)<br>L: 0.82	1.62 (1.07, 2.46)<br>NL: 0.01<br>C: 0.02	1.01 (0.83, 1.23)<br>L: 0.91
**S3**	1.11 (0.90, 1.38)<br>L: 0.34	1.04 (0.86, 1.26)<br>L: 0.66	1.57 (1.11, 2.24)<br>NL: 0.008<br>C: 0.01	1.05 (0.85, 1.29)<br>L: 0.66
**S4**	1.04 (0.88, 1.23)<br>L: 0.64	1.06 (0.91, 1.22)<br>L: 0.47	1.53 (1.12, 2.08)<br>NL: 0.03<br>C: 0.01	1.09 (0.90, 1.31)<br>L: 0.38
**S5**	1.00 (0.90, 1.12)<br>L: 0.94	1.04 (0.95, 1.14)<br>L: 0.43	1.15 (1.03, 1.28)<br>L: 0.02	1.07 (0.95, 1.20)<br>L: 0.29

KEY

U=Minimally adjusted analysis
A=Fully adjusted analysis
S1=Missing province-years received $0·04 in annual per capita funding
S2=Missing province-years received $0·23 in annual per capita funding
S3=Missing province-years received $0·32 in annual per capita funding
S4=Missing province-years received $0·56 in annual per capita funding
S5=Missing province-years received $0·93 in annual per capita funding
S6=Inverse probability weighting
■ Funding associated with improved health outcomes
■ Funding significantly associated with improved health outcomes
■ Concave relationship between funding & health outcomes
■ Significant concave relationship between funding & health outcomes
■ Funding associated with worse health outcomes
■ Funding significantly associated with worse health outcomes
☐ Funding not associated with health outcomes

© 2019 Barnhart DA et al. *JAMA Network Open.*

eTable 6. Association Between Dichotomized Pre- vs Post-PEPFAR Funding for PMTCT and Infant Mortality Stratified by Maternal HIV Status

Analysis conducted among infants among infants with known maternal HIV status based on HIV testing conducted in the 2003 and 2008 surveys.

	Maternal HIV Status		Test for interaction between PEPFAR funding and HIV status
	HIV negative n/N=214/3803	HIV positive n/N=45/369	
	RR, (95% CI)	RR, (95% CI)	
**No lag**	0.32 (0.13, 0.77)	0.63 (0.24, 1.67)	0.09
**1 year lag**	0.53 (0.33, 0.86)	1.00 (0.43, 2.30)	0.15
**2 year lag**	1.08 (0.62, 1.90)	1.88 (0.73, 4.85)	0.28
**3 year lag**	0.77 (0.40, 1.46)	1.47 (0.49, 4.37)	0.33

© 2019 Barnhart DA et al. *JAMA Network Open.*

eReferences

1. Fitzmaurice G, Laird N, Ware J. Applied Longitudinal Analysis. 2 ed. Hoboken, New Jersey: John Wiley & Sons, Inc; 2011.
2. ICF International. Demographic and Health Survey Sampling and Household Listing Manual. Calverton, Maryland, U.S.A.: ICF International; 2012.
3. Wacholder S. Binomial regression in GLIM: estimating risk ratios and risk differences. Am J Epidemiol 1986;123:174-84.
4. Zou G. A modified poisson regression approach to prospective studies with binary data. Am J Epidemiol 2004;159:702-6.
5. Durrleman S, Simon R. Flexible regression models with cubic splines. Stat Med 1989;8:551-61.
6. The SAS GLMCURV9 Macro. 2014. (https://www.hsph.harvard.edu/donna-spiegelman/software/glmcurv9/.)
7. Staveteig S, Mallick L. Intertemporal comparisons of poverty and wealth with DHS data: A harmonized asset index approach. Rockville, Maryland, USA: ICF International; 2014.
8. Seaman SR, White IR. Review of inverse probability weighting for dealing with missing data. Stat Methods Med Res 2013;22:278-95.

© 2019 Barnhart DA et al. *JAMA Network Open.*
